# Risk factors for early-onset radiodermatitis in patients with locally advanced breast cancer: a comparative analysis of volumetric modulated arc therapy and intensity-modulated radiotherapy

**DOI:** 10.1007/s12282-025-01762-y

**Published:** 2025-08-27

**Authors:** Chih-Chieh Chang, Po-Wei Huang, Jang-Chun Lin, Jo-Ting Tsai, Shih-Ming Hsu

**Affiliations:** 1 Department of Radiation Oncology, Shuang Ho Hospital, Taipei Medical University, New Taipei, 235 Taiwan, ROC; 2 Graduate Institute of Clinical Medicine, College of Medicine, Taipei Medical University, Taipei, 110 Taiwan, ROC; 3 Department of Radiology, School of Medicine, College of Medicine, Taipei Medical University, Taipei, 110 Taiwan, ROC; 4 Medical Physics and Radiation Measurements Laboratory, National Yang Ming Chiao Tung University, Taipei, Taiwan, ROC; 5 Department of Biomedical Imaging and Radiological Sciences, National Yang Ming Chiao Tung University, No.155, Sec.2, Li-Nong St., Beitou District, Taipei, 112 Taiwan, ROC

**Keywords:** Breast cancer, Radiodermatitis, Intensity-modulated radiotherapy, Volumetric modulated arc therapy, Risk factors

## Abstract

**Background:**

Radiodermatitis is the predominant acute toxicity in locally advanced breast cancer (BC) radiotherapy. Early-onset radiodermatitis substantially increases the risk of high-grade injury and potential treatment interruption. This study aimed to identify risk factors for early-onset radiodermatitis by analyzing patient characteristics and dose-distribution profiles from step-and-shoot intensity-modulated radiotherapy (IMRT) and volumetric-modulated arc therapy (VMAT).

**Methods:**

This retrospective analysis included 128 women with locally advanced unilateral BC who received postoperative IMRT or VMAT. Patient demographics, treatment parameters, and three-dimensional dose–volume metrics were extracted. Early-onset radiodermatitis was defined as CTCAE v5.0 grade ≥ 1 developing before the 20th treatment fraction. Univariable and multivariable logistic regression identified independent risk factors, and dosimetric variables were compared between IMRT and VMAT.

**Results:**

Early-onset radiodermatitis was observed in 35 of 128 patients (27.3%). Four independent predictors were identified: treatment technique (IMRT versus VMAT: odds ratio [OR] 4.25; 95% confidence interval [CI] 1.30–13.94; *p* = 0.017), left-sided irradiation (OR 20.98; 95% CI 2.39–184.27; *p* = 0.006), heart *V*_5_ (OR 0.85 per 1% increase; 95% CI 0.74–0.97; *p* = 0.012), and PTV_Breast_ volume (OR 1.002 per cc; 95% CI 1.000–1.004; *p* = 0.023). VMAT provided superior target coverage and reduced high-dose exposure to the heart and ipsilateral lung (*V*_40_), whereas IMRT better limited low-dose spill to the contralateral breast and lung and to the ipsilateral lung (*V*_5_).

**Conclusions:**

IMRT, left-sided irradiation, low heart *V*_5_, and large PTV_Breast_ volume were independent risk factors for early-onset radiodermatitis. Awareness of these factors can guide prophylactic skin care and adaptive planning. While VMAT provides advantages in target coverage and cardiac/high-dose lung sparing against IMRT’s superior control of low-dose exposure to normal tissues.

## Introduction

According to the Global Cancer Observatory: Cancer estimates [1], breast cancer (BC) was the predominant type of cancer in women in 2022, with 2.3 million new cases reported in that year, constituting 11.6% of all cancer diagnoses. BC results in approximately 666,000 deaths every year, accounting for 6.9% of all cancer-related deaths worldwide. Currently, the primary treatment for BC involves surgical intervention in the form of either modified radical mastectomy (MRM) or breast-conserving surgery (BCS), followed by radiotherapy. Postoperative radiotherapy effectively reduces the risk of postoperative local recurrence and improves the rate of survival [2, 3]. For patients with locally advanced BC, which is characterized by metastatic lymph nodes, treatment strategies may also include radiation targeting the axillary lymph nodes, supraclavicular fossa (SCF) lymph nodes, and internal mammary (lymph) node (IMN) chain [4–6].

In patients with BC, radiotherapy can cause side effects such as fatigue, appetite loss, skin reactions or radiodermatitis, sore throat, radiation pneumonitis, cardiotoxicity, local tissue fibrosis, and lymphedema [7–9]. Among these, radiodermatitis is the predominant acute side effect of radiotherapy [10]. Symptoms of radiodermatitis range from mild erythema, hair loss, and dry desquamation to severe wet desquamation and ulcers. In addition, severe skin reactions due to radiotherapy can reduce patients’ quality of life and interrupt treatment [11]. Xie et al. classified the risk factors for radiodermatitis into patient-related and treatment-related factors [12]. Patient-related factors include age, obesity, breast size, and smoking habits, and treatment-related factors include radiation dose to the skin, treated area, bolus usage, and type of chemotherapeutic agent. However, very few studies have explored risk factors for early-onset radiodermatitis in patients with BC. Early-onset cases increase the risk of severe skin toxicity and treatment interruption. Moreover, the literature has typically presented data from patients across all stages of BC rather than data from specific analyses for those with locally advanced BC. Thus, this retrospective study identified key risk factors for early-onset radiodermatitis in patients with locally advanced BC.

The studies determining the maximum allowable doses received by the heart and lung during BC radiotherapy have focused on minimizing risks to these vital organs while maintaining treatment efficacy [13, 14]. Meeting dose limits is highly challenging in planning radiotherapy for locally advanced BC. However, radiotherapy techniques have improved from three-dimensional conformal radiotherapy to advanced techniques such as intensity-modulated radiotherapy (IMRT) and volumetric modulated arc therapy (VMAT), which have addressed this challenge [15–17]. The primary advantage of VMAT over IMRT lies in its greater treatment delivery efficiency [18]. In addition, VMAT ensures minimal exposure of vital organs to radiation while matching the tumor dose coverage and plan quality of IMRT [19, 20]. However, the comparative studies that have been conducted on this topic have often involved simulated rather than actual treatment plans. In contrast, the present study compared dosimetric parameters, including those related to target areas and organs at risk (OARs), between IMRT and VMAT.

In this study, we analyzed risk factors for early-onset radiodermatitis in patients with locally advanced BC treated using IMRT and VMAT and compared dosimetric parameters between these two techniques. The results can guide the development of future treatment strategies.

## Methods

### Patient selection and data collection

This retrospective study included women with locally advanced unilateral BC who received postoperative radiotherapy targeting the breast/chest wall and regional lymph nodes. Patients receiving radiotherapy for the first time who had completed the full course of treatment and were treated exclusively with either IMRT or VMAT were deemed eligible for inclusion in this study. The selection of radiotherapy technique was based on the treatment era within the study cohort period (2016–2020). To reduce patient treatment time, our institution began to implement VMAT for locally advanced BC patients in 2018. Consequently, patients treated before 2018 predominantly received IMRT, while those treated thereafter predominantly received VMAT. A total of 128 patients were included. This study was approved by the Joint Institutional Review Board of Taipei Medical University (Permit number: N201912025). The following data were collected: patient demographics, radiodermatitis severity (assessed using the Common Terminology Criteria of Adverse Events) [21], treatment fractions at which radiodermatitis occurred, treatment plans, and dosimetric parameters related to target areas and OARs. Additional treatment-related factors, such as the use of cone beam computed tomography (CBCT)-guided therapy and Deep Inspiration Breath Hold (DIBH) techniques, were also recorded.

### Radiotherapy plan

Radiotherapy was delivered in two phases. In Phase I, radiation was delivered to the entire breast/chest wall and lymph nodes using one of two standard institutional regimens, either 50 Gy in 25 fractions or 50.4 Gy in 28 fractions. The selection of the regimen was primarily guided by the type of surgery. The 50 Gy regimen was used for nearly all patients who had undergone BCS (55 of 56, 98.2%), while the 50.4 Gy regimen was the more common choice for patients after MRM (48 of 72, 66.7%). The 50 Gy regimen was also used for a substantial portion of MRM cases at the physician's discretion. In Phase II, tumor boost radiation (10 Gy) was delivered to the tumor bed or postoperative scars (selected at the clinician’s discretion on the basis of clinical necessity) in five fractions.

The patients were treated in a hands-up position by using a vacuum immobilization pad. CT images (slice thickness: 5 mm) were acquired using the Brilliance Big Bore CT scanner (Philips, Cambridge, MA, USA). Contouring and treatment planning were performed using the Pinnacle^3^ treatment planning system (version 9.8; Philips). Clinical target volumes and OARs [spinal cord, esophagus, ipsilateral and contralateral lungs, heart, left ventricle (LV), and contralateral breast] were delineated following the atlas published by the Radiation Therapy Oncology Group [22]. The planning target volume (PTV) was defined as a 5-mm isotropic expansion from the clinical target volume, with a 3-mm contraction from the skin surface. The PTV for the breast (PTV_Breast_) included the entire breast or chest wall. Whether axillary lymph nodes and IMNs within PTV_Breast_ were included was determined on the basis of clinical necessity. The PTV for the SCF (PTV_SCF_) covered the SCF region. Treatment was delivered using the Elekta Synergy system (Elekta limited, Crawley, UK), equipped with a 1-cm-wide multileaf collimator and operating at a therapeutic energy of 6 MV.

DIBH was implemented for patients with left-sided BC treated with the VMAT technique who could follow breathing guidance and maintain a stable breath-hold for over 30 s.

**Fig. 1:**
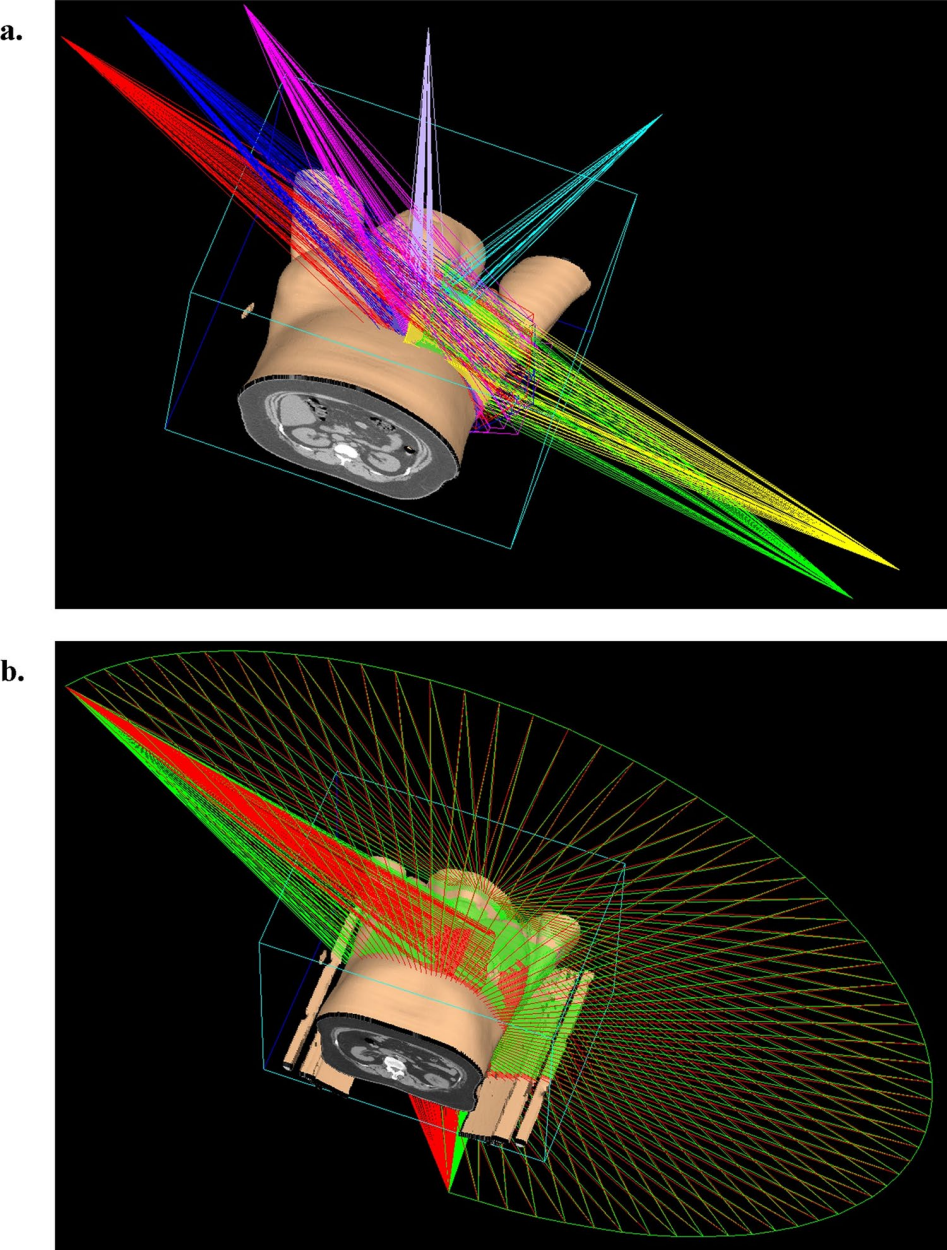
Beam arrangements for breast cancer radiotherapy. (**a**) Plan for seven-field IMRT. (**b**) Plan for two three-quarter arc VMAT. *IMRT* intensity-modulated radiotherapy, *VMAT* volumetric modulated arc therapy

IMRT involved 7–9 fields, whereas VMAT involved 2 ipsilateral VMAT fields spanning approximately 240° (2/3-circle arcs). The arrangement of IMRT fields and the configuration of VMAT arcs are depicted in Fig. 1. The radiation dose was calculated using the adaptive convolution algorithm, with an isotropic resolution of 3 mm. The aim of the radiotherapy plan was to achieve a PTV V_95%_ (percentage of the target volume that receives at least 95% of the prescribed radiation dose) of > 95% and a PTV *D*_max_ (maximum dose) of < 110% of the prescribed dose. Dose limits for OARs were set following the Quantitative Analyses of Normal Tissue Effects in the Clinic guidelines [23], with exposure minimized as much as possible.

### Statistical analysis of risk factors for early-onset radiodermatitis

To identify key risk factors for early-onset radiodermatitis, the patients were stratified into two groups by the timing of radiodermatitis onset: an early-onset group (*n* = 35), comprising patients who developed radiodermatitis before the 20th treatment fraction, and a non/late-onset group (*n* = 93), comprising patients who did not develop radiodermatitis or developed it after the 20th treatment fraction. This threshold was selected based on institutional data, corresponding to approximately the earliest 30% of radiodermatitis onset times, which aligns with the 20th treatment fraction (approximately two-thirds of a 30-fraction course, including boost).

Univariate analysis was performed to identify potential risk factors for early-onset radiodermatitis—for example, patient demographics, radiotherapy plan, and dosimetric parameters related to target areas and OARs. Tables 1 and 2 present a summary of 65 potential risk factors. Categorical variables were compared between the early-onset and non/late-onset groups by using the Pearson chi-square test or Fisher exact test (if any cell counts were < 5). Continuous variables were analyzed using independent-samples *t* tests; the nonparametric Mann–Whitney *U* test was used when the assumption of normality was violated.

**Table 1:** Results of univariate analysis of demographic and radiotherapy plan–related risk factors for early-onset radiodermatitis

Variables	Category	None/late-onset group (*n* = 93)	Early-onset group (*n* = 35)	*p* value
Age at enrolment (years)		57.45 ± 9.92	55.46 ± 11.99	0.341
BMI (kg/m^2^)		24.87 ± 4.5	26.5 ± 5.45	0.144
Monitor unit		660.44 ± 178.89	696.65 ± 212.55	0.453
Tumor stage^a^	T0–T1	33 (35.48%)	10 (28.57%)	0.461
	T2–T4	60 (64.52%)	25 (71.43%)	
Nodal stage^a^	N0–N1	71 (76.34%)	25 (71.43%)	0.567
	N2–N4	22 (23.66%)	10 (28.57%)	
ER	Negative	19 (20.43%)	6 (17.14%)	0.676
	Positive	74 (79.57%)	29 (82.86%)	
PR	Negative	27 (29.03%)	8 (22.86%)	0.485
	Positive	66 (70.97%)	27 (77.14%)	
Her2	Negative	70 (75.27%)	30 (85.71%)	0.203
	Positive	23 (24.73%)	5 (14.29%)	
Surgery	BCS	36 (38.71%)	20 (57.14%)	0.061
	MRM	57 (61.29%)	15 (42.86%)	
Implant	W/O	80 (86.02%)	34 (97.14%)	0.110
	W/	13 (13.98%)	1 (2.86%)	
Chemotherapy category	None	2 (2.15%)	3 (8.57%)	0.143
	Taxane	79 (84.95%)	30 (85.71%)	
	Others	12 (12.9%)	2 (5.71%)	
HT	W/O	87 (93.55%)	30 (85.71%)	0.171
	W/	6 (6.45%)	5 (14.29%)	
Target therapy	W/O	72 (77.42%)	28 (80%)	0.753
	W/	21 (22.58%)	7 (20%)	
Treatment site	Left breast	45 (48.39%)	20 (57.14%)	0.377
	Right breast	48 (51.61%)	15 (42.86%)	
Prescribed dose	50.00 Gy	52 (55.91%)	27 (77.14%)	0.028
	50.40 Gy	41 (44.09%)	8 (22.86%)	
Treatment technique	IMRT	43 (46.24%)	20 (57.14%)	0.271
	VMAT	50 (53.76%)	15 (42.86%)	
DIBH	W/O	83 (89.25%)	30 (85.71%)	0.552
	W/	10 (10.75%)	5 (14.29%)	
CBCT	W/O	39 (41.94%)	13 (37.14%)	0.623
	W/	54 (58.06%)	22 (62.86%)	
Field included axilla node	W/O	36 (38.71%)	14 (40%)	0.894
	W/	57 (61.29%)	21 (60%)	
Field included IMN	W/O	87 (93.55%)	31 (88.57%)	0.460
	W/	6 (6.45%)	4 (11.43%)	
Bolus	W/O	51 (54.84%)	23 (65.71%)	0.267
	W/	42 (45.16%)	12 (34.29%)	

*W/* with, *W/O* without, *BCS* breast-conserving surgery, *MRM* modified radical mastectomy, *ER* estrogen receptor, *HER2* human epidermal growth factor receptor 2, *PR* progesterone receptor, *IMRT* intensity-modulated radiation therapy, *VMAT* volumetric modulated arc therapy, *CBCT* cone beam computed tomography, *IMN* internal mammary nodes, *DIBH* deep inspiration breath hold radiotherapy, *HT* hormonal therapy, *BMI* body mass index

^a^Pathology stage

**Table 2:** Results of the univariate analysis of dosimetric parameter–related risk factors for early-onset radiodermatitis

Targets/organs	Variable (unit)	None/late-onset group (*n* = 93)	Early-onset group (*n* = 35)	*p* value
PTV_Breast_	Volume (cc)	564.8 ± 237.13	742.3 ± 347.72	0.002
	*D*_max_ (Gy)	55.01 ± 0.46	54.87 ± 0.5	0.205
	*D*_mean_ (Gy)	51.79 ± 0.29	51.69 ± 0.39	0.348
	*V*_105%_ (%)	29.61 ± 11.77	28.29 ± 11.05	0.567
	*V*_95%_ (%)	96.96 ± 1.16	97.04 ± 1.43	0.403
	CI_PTVBreast_	0.89 ± 0.03	0.89 ± 0.04	0.636
	HI_PTVBreast_	0.16 ± 0.03	0.16 ± 0.06	0.469
PTV_SCF_	Volume (cc)	91.22 ± 27.32	104.26 ± 36.19	0.045
	*D*_max_ (Gy)	54.26 ± 0.76	54.15 ± 0.68	0.291
	*D*_mean_ (Gy)	51.44 ± 0.53	51.29 ± 0.56	0.161
	*V*_105%_ (%)	19.82 ± 15.11	16.75 ± 14.98	0.291
	*V*_95%_ (%)	95.73 ± 3.6	95.55 ± 3.91	0.904
	CI_PTVSCF_	0.87 ± 0.07	0.87 ± 0.07	0.883
	HI_PTVSCF_	0.17 ± 0.1	0.17 ± 0.1	0.892
Heart	Volume (cc)	446.91 ± 108.89	450.15 ± 95.38	0.619
	*D*_max_ (Gy)	28.27 ± 20.11	28.22 ± 20.24	0.720
	*D*_mean_ (Gy)	3.18 ± 1.92	2.9 ± 1.59	0.356
	*V*_40_ (%)	0.73 ± 1.59	0.44 ± 0.75	0.745
	*V*_25_ (%)	1.95 ± 3.07	1.51 ± 1.97	0.889
	*V*_10_ (%)	4.4 ± 5.56	4 ± 4.4	0.961
	*V*_5_ (%)	9.85 ± 9.25	8.67 ± 7.81	0.437
LV	Volume (cc)	139.88 ± 29.14	138.33 ± 27.12	0.875
	*D*_max_ (Gy)	22.62 ± 22	23.71 ± 21.41	0.497
	*D*_mean_ (Gy)	3.44 ± 2.88	3.36 ± 2.51	0.734
	*V*_40_ (%)	0.77 ± 2.01	0.49 ± 1.02	0.985
	*V*_25_ (%)	2.53 ± 4.13	2.24 ± 3.13	0.910
	*V*_10_ (%)	6.15 ± 8.08	5.99 ± 6.85	0.684
	*V*_5_ (%)	12.45 ± 14.29	12.38 ± 12.04	0.874
Ipsilateral lung	Volume (cc)	1297.2 ± 328.35	1222.73 ± 357.47	0.212
	*D*_mean_ (Gy)	12.85 ± 1.51	12.26 ± 2.29	0.281
	*V*_40_ (%)	11.72 ± 2.88	10.79 ± 3.29	0.120
	*V*_30_ (%)	17.89 ± 3.16	16.79 ± 4.17	0.270
	*V*_20_ (%)	24.41 ± 3.53	23.22 ± 5.28	0.632
	*V*_10_ (%)	34.64 ± 3.99	33.33 ± 6.28	0.566
	*V*_5_ (%)	47.79 ± 4.92	46.33 ± 7.62	0.167
Contralateral lung	Volume (cc)	1263.93 ± 406.12	1256.44 ± 389.84	0.942
	*D*_mean_ (Gy)	1.19 ± 0.62	1.08 ± 0.63	0.443
	*V*_10_ (%)	0.12 ± 0.27	0.13 ± 0.44	0.120
	*V*_5_ (%)	1.7 ± 2.25	1.29 ± 2.1	0.126
	*V*_2_ (%)	16.31 ± 14.62	13.02 ± 14.23	0.175
Contralateral breast	Volume (cc)	582.61 ± 273.96	732.21 ± 354.74	0.013
	*D*_mean_ (Gy)	3.23 ± 1.65	2.56 ± 1.45	0.049
	*V*_5_ (%)	18.09 ± 12.12	13.75 ± 11.06	0.064
	*V*_2_ (%)	41.6 ± 22.71	35.69 ± 25.5	0.182

*PTV* planning target volume, *LV* left ventricle, *V*_*x%*_ the proportions of the irradiation volume with doses of *x*% of prescribed dose to the specific target or organ volume, *V*_*x*_ the proportions of the irradiation volume with a dose of *x* Gy to the specific target or organ volume, *CI*_*x*_ Conformity index of target *x*, *HI*_*x*_ homogeneity index of target *x*

All variables were entered into a multivariate binomial logistic regression model. To address multicollinearity, variables with variance inflation factors of > 10 were sequentially removed until no variables exhibited excessive collinearity. Subsequently, backward stepwise selection was performed; we started with the full model and iteratively removed variables that did not significantly contribute to the model’s predictive ability.

Patients were further stratified by cancer side (left or right breast) and treatment technique (VMAT/IMRT). Among the patients with left-sided BC, 26 received IMRT and 39 VMAT. Among the patients with right-sided BC, 37 received IMRT and 26 VMAT. Dosimetric differences between these groups were analyzed using two-tailed independent-samples *t* tests or the Mann–Whitney *U* test, as appropriate. All statistical analyses were performed using SPSS (version 24.0; SPSS Inc., Chicago, IL, USA). Significance was set at *p* < 0.05.

## Results

### Characteristics of the study cohort

Among the patients, 101 (78.9%) had Grade 1 radiodermatitis, whereas 11 (8.6%) had Grade 2 radiodermatitis. No patient had Grade ≥ 3 radiodermatitis. The mean number of treatment fractions leading to Grade 1 radiodermatitis was significantly lower in the early-onset group (18.8 ± 1.2) than in the non/late-onset group (25.3 ± 1.9). The likelihood of Grade 1 radiodermatitis progressing to Grade 2 radiodermatitis was 3.2-fold higher in the early-onset group than in the non/late-onset group (6/35 vs. 5/93), but this difference was nonsignificant (*p* = 0.069).

### Risk factors for early-onset radiodermatitis

The univariate analysis revealed significant associations between several factors and the development of early-onset radiodermatitis (Tables 1 and 2). Among the categorical variables, only prescribed dose was significantly associated with the development of early-onset radiodermatitis; the risk of early-onset radiodermatitis was elevated in patients receiving 50 Gy of radiation (*p* = 0.028). Among the continuous variables, large PTV_Breast_ (*p* = 0.002), large PTV_SCF_ (*p* = 0.045), and large contralateral breast volume (*p* = 0.013) were associated with higher risks of early-onset radiodermatitis. Conversely, a lower contralateral breast *D*_mean_ (average dose received by the contralateral breast; *p* = 0.049) was associated with a higher risk of early-onset radiodermatitis.

After clinical evaluation and collinearity assessment, 37 variables were included in the multivariate logistic regression analysis: age at enrollment, treatment site, body mass index, estrogen receptor, progesterone receptor, human epidermal growth factor receptor 2, surgery type, implant presence, chemotherapy category, target therapy, hormone therapy, treatment field including axillary lymph nodes, treatment field including IMNs, treatment technique, DIBH, bolus usage, monitor units, cone beam CT, PTV_Breast_ *D*_max_, PTV_Breast_ *D*_mean_, PTV_Breast_ *V*_95%_, PTV_Breast_ volume, PTV_SCF_ *D*_max_, PTV_SCF_ *V*_95%_, PTV_SCF_ volume, CI_PTVBreast_, CI_PTVSCF_, HI_PTVBreast_, HI_PTVSCF_, heart *V*_40_, heart *V*_5_, LV *V*_25_, ipsilateral lung *V*_40_, ipsilateral lung *V*_5_, contralateral lung *V*_10_, contralateral lung *V*_5_, and contralateral breast *V*_5_. For clarity, key dosimetric acronyms are defined as follows: conformity index (CI) and homogeneity index (HI). For dose–volume metrics, *V*_x_ represents the proportions of the irradiation volume with a dose of *x* Gy.

The results of the multivariate logistic regression analysis are summarized in Table 3. Six factors were included in the final model. The overall model was statistically significant compared with the null model (*χ*^2^ = 27.075; *p* < 0.001). An increase in the risk of early-onset radiodermatitis was significantly associated with left-sided BC (odds ratio [OR] = 20.98; *p* < 0.01), large PTV_Breast_ volume (OR = 1.002; *p* = 0.023), and IMRT (OR = 4.251; *p* = 0.017). By contrast, a high heart *V*_5_ value (OR = 0.846; *p* = 0.012) was associated with a lower risk of early-onset radiodermatitis. No significant association was observed between the risk of early-onset radiodermatitis and PTV_SCF_ volume (*p* = 0.063) or PTV_SCF_ *D*_max_ (*p* = 0.097).

**Table 3:** Results of the multivariate logistic regression analysis of risk factors for early-onset radiodermatitis

	OR	95% C.I. for OR		
		Lower	Upper	*p* value
Treatment technique (IMRT vs. VMAT)	4.251	1.296	13.944	0.017
Treatment site (left breast vs. right breast)	20.980	2.389	184.271	0.006
Heart *V*_5_ (%)	0.846	0.742	0.965	0.012
PTV_Breast_ volume (cc)	1.002	1.000	1.004	0.023
PTV_SCF_ volume (cc)	1.014	0.999	1.030	0.063
PTV_SCF_ *D*_max_ (Gy)	0.526	0.247	1.123	0.097

*OR* odds ratio, *C.I.* confidence interval, *PTV* planning target volume, *V*_*x*_ the proportions of the irradiation volume with a dose of *x* Gy to the specific target or organ volume, *IMRT* intensity-modulated radiation therapy, *VMAT* volumetric modulated arc therapy

### Dosimetric comparisons between IMRT and VMAT

Table 4 presents the dosimetric parameters for the target volumes. For patients with left-sided BC, VMAT demonstrated significantly superior target coverage for both PTV_Breast_ and PTV_SCF_, as evidenced by higher V_95%_ values (*p* < 0.001 for both). VMAT also yielded significantly better conformity (higher CI) and homogeneity (lower HI) for both target volumes (*p* < 0.05 for all). For patients with right-sided BC, VMAT also achieved significantly superior V_95%_ coverage for both PTV_Breast_ (*p* = 0.004) and PTV_SCF_ (*p* < 0.001). While conformity and homogeneity for the PTV_SCF_ were also significantly better with VMAT (*p* < 0.001), no statistically significant differences were observed for these metrics for the PTV_Breast_ (*p* > 0.1 for both CI and HI).

**Table 4:** Dosimetric parameters related to target areas in patients with left-sided or right-sided BC receiving IMRT or VMAT

Target	Variable (unit)	Left-sided BC			Right-sided BC		
		IMRT (*n* = 26)	VMAT (*n* = 39)	*p* value	IMRT (*n* = 37)	VMAT (*n* = 26)	*p* value
PTV_Breast_	Volume (cc)	600.31 ± 402.08	664.35 ± 302.89	0.073	585.26 ± 206.45	589.79 ± 186.92	0.929
	*D*_max_ (Gy)	54.99 ± 0.42	55.12 ± 0.52	0.244	54.8 ± 0.41	54.98 ± 0.49	0.066
	*D*_mean_ (Gy)	51.62 ± 0.42	51.86 ± 0.3	0.010	51.7 ± 0.28	51.84 ± 0.24	0.051
	*V*_105%_ (%)	26.91 ± 12.01	33.84 ± 9.27	0.011	25.16 ± 12.64	30.5 ± 10.38	0.081
	V_95%_ (%)	95.83 ± 1.29	97.18 ± 1.06	0.000	97.04 ± 1.03	97.76 ± 0.84	0.004
	CI_PTVBreast_	0.87 ± 0.04	0.89 ± 0.04	0.043	0.89 ± 0.03	0.9 ± 0.03	0.281
	HI_PTVBreast_	0.2 ± 0.07	0.16 ± 0.03	0.007	0.15 ± 0.03	0.14 ± 0.02	0.124
PTV_SCF_	Volume (cc)	83.86 ± 27.06	100.82 ± 38.82	0.069	95.36 ± 23.26	95.85 ± 26.78	0.938
	*D*_max_ (Gy)	54.73 ± 0.45	53.82 ± 0.76	0.000	54.55 ± 0.47	53.88 ± 0.72	0.000
	*D*_mean_ (Gy)	51.27 ± 0.6	51.49 ± 0.43	0.095	51.37 ± 0.62	51.46 ± 0.51	0.518
	*V*_105%_ (%)	25.93 ± 13.94	12.4 ± 13.57	0.000	24.84 ± 13.77	13.56 ± 14.45	0.001
	*V*_95%_ (%)	92.58 ± 3.28	97.73 ± 1.94	0.000	94.35 ± 3.95	97.62 ± 2.57	0.000
	CI_PTVSCF_	0.82 ± 0.06	0.91 ± 0.06	0.000	0.85 ± 0.07	0.91 ± 0.05	0.000
	HI_PTVSCF_	0.24 ± 0.08	0.12 ± 0.06	0.000	0.2 ± 0.1	0.13 ± 0.09	0.000

*BC* breast cancer, *PTV* planning target volume, *Vx%* the proportions of the irradiation volume with doses of *x*% of prescribed dose to the specific target or organ volume, *CIx* conformity index of target *x*, *HIx* homogeneity index of target *x*, *IMRT* intensity-modulated radiation therapy, *VMAT* volumetric modulated arc therapy

The dosimetric effects of IMRT and VMAT on OARs are summarized in Table 5. In patients with left-sided BC, VMAT significantly reduced exposure to the heart, as evidenced by reduced *D*_max_, *V*_40_, *V*_25_, and *V*_10_ (*p* < 0.01 for all). Similar reductions were observed for the LV, as evidenced by reduced *D*_max_, *D*_mean_, *V*_40_, *V*_25_, and *V*_10_ (*p* < 0.05 for all), suggesting cardioprotective benefits of VMAT. In patients with right-sided BC, IMRT achieved superior sparing of the heart (reduced *D*_mean_, *p* < 0.01) and LV (reduced *D*_max_ and *D*_mean_, *p* < 0.01). Regarding lung dosimetry, VMAT reduced the high-dose burden to the ipsilateral lung, as reflected by significantly lower *V*_40_ (*p* < 0.01), yet it produced a modest increase in low-dose exposure (*V*_5_, *p* < 0.01) in both left- and right-sided BC patients. By contrast, IMRT afforded better contralateral lung sparing, with significantly lower mean dose, *V*_5_, and *V*_2_ (all *p* < 0.01). In addition, compared with VMAT, IMRT significantly reduced exposure to the contralateral breast (reduced mean dose, *V*_5_, and *V*_2_, *p* < 0.01) in both patients with left-sided BC and those with right-sided BC.

**Table 5:** Dosimetric parameters related to organs at risk in patients with left-sided or right-sided BC receiving IMRT or VMAT

Critical organ	Variable (unit)	Left-sided BC			Right-sided BC		
		IMRT (*n* = 26)	VMAT (*n* = 39)	*p* value	IMRT(*n* = 37)	VMAT (*n* = 26)	*p* value
Heart	Volume (cc)	458.94 ± 129.46	453.34 ± 102.61	0.847	435.5 ± 78.34	445.84 ± 118.36	0.699
	*D*_max_ (Gy)	50.23 ± 3.08	44.6 ± 8.56	0.000	9.36 ± 6.02	8.65 ± 5.05	0.883
	*D*_mean_ (Gy)	4.98 ± 1.7	4.38 ± 0.79	0.105	1.18 ± 0.29	2.06 ± 0.53	0.000
	*V*_40_ (%)	2.43 ± 2.29	0.51 ± 0.59	0.000			
	*V*_25_ (%)	5.68 ± 3.53	2.21 ± 1.54	0.000			
	*V*_10_ (%)	11.45 ± 4.86	6.27 ± 2.91	0.000	0.15 ± 0.39	0.07 ± 0.27	0.385
	*V*_5_ (%)	18.17 ± 5.32	16.46 ± 5.55	0.220	1.42 ± 2.33	2.03 ± 2.89	0.348
LV	Volume (cc)	131.73 ± 30.89	141.5 ± 25.26	0.168	140.18 ± 26.37	143.09 ± 33.45	0.933
	*D*_max_ (Gy)	47.5 ± 4.77	40.52 ± 10.02	0.002	1.35 ± 0.64	2.62 ± 0.99	0.000
	*D*_mean_ (Gy)	6.44 ± 2.33	5.39 ± 1.22	0.049	0.63 ± 0.13	1.43 ± 0.44	0.000
	*V*_40_ (%)	2.64 ± 3.18	0.51 ± 0.79	0.000			
	*V*_25_ (%)	7.33 ± 5.04	3.16 ± 2.59	0.000			
	V_10_ (%)	15.83 ± 7.18	9.48 ± 5.27	0.000			
	*V*_5_ (%)	26.34 ± 8.55	23.24 ± 8.22	0.147			
Ipsilateral lung	Volume (cc)	1048.27 ± 221.08	1330.45 ± 448.32	0.010	1347.93 ± 258.53	1323.82 ± 238.64	0.708
	*D*_mean_ (Gy)	12.19 ± 2.63	12.83 ± 1.44	0.659	12.74 ± 1.71	12.91 ± 1.1	0.657
	*V*_40_ (%)	12.38 ± 3.8	10.54 ± 2.69	0.006	12.38 ± 2.96	10.63 ± 1.98	0.011
	*V*_30_ (%)	17.75 ± 4.85	17.15 ± 3.16	0.164	17.99 ± 3.38	17.51 ± 2.41	0.536
	*V*_20_ (%)	23.46 ± 6	24.05 ± 3.46	0.820	24.23 ± 3.96	24.57 ± 2.81	0.707
	*V*_10_ (%)	32.6 ± 7.16	34.68 ± 3.71	0.538	34.46 ± 4.4	35.1 ± 3.12	0.527
	*V*_5_ (%)	42.94 ± 8.27	49.41 ± 4.39	0.000	46.88 ± 4.72	49.53 ± 2.94	0.008
Contralateral lung	Volume (cc)	1260.45 ± 260	1593.12 ± 476.29	0.005	1066.06 ± 219.42	1045.14 ± 234.91	0.665
	*D*_mean_ (Gy)	0.66 ± 0.3	1.8 ± 0.38	0.000	0.62 ± 0.27	1.49 ± 0.33	0.000
	*V*_10_ (%)	0.09 ± 0.23	0.13 ± 0.23	0.520	0.1 ± 0.39	0.18 ± 0.4	0.202
	*V*_5_ (%)	0.47 ± 0.76	2.83 ± 2.43	0.000	0.57 ± 1.17	2.29 ± 2.73	0.000
	*V*_2_ (%)	4.51 ± 6.55	30.53 ± 11.54	0.000	3.21 ± 3.62	20.98 ± 8.71	0.000
Contralateral breast	Volume (cc)	732.7 ± 375.28	679.09 ± 346.41	0.556	553.96 ± 198.61	529.94 ± 237.85	0.665
	*D*_mean_ (Gy)	1.92 ± 1.12	3.95 ± 1.17	0.000	2.14 ± 1.17	4.11 ± 1.73	0.000
	*V*_5_ (%)	10.02 ± 7.88	22.29 ± 9.91	0.000	11.3 ± 9.96	23.69 ± 13.52	0.000
	*V*_2_ (%)	19.67 ± 11.44	58.02 ± 15.72	0.000	23.3 ± 12.57	56.98 ± 20.85	0.000

*BC* breast cancer, *LV* left ventricle, *Vx* the proportions of the irradiation volume with a dose of *x* Gy to the specific target or organ volume, *IMRT* intensity-modulated radiation therapy, *VMAT* volumetric modulated arc therapy

## Discussion

We identified key risk factors for early-onset radiodermatitis in patients with locally advanced unilateral BC. Our univariate analysis revealed that the risk of early-onset radiodermatitis was associated with prescribed dose, PTV_Breast_, PTV_SCF_, contralateral breast volume, and contralateral breast *D*_mean_. Of note, the association with a prescribed dose of 50 Gy was one of these significant findings. However, this factor was not included in the multivariable model due to its strong collinearity with surgical type and PTV_Breast_ volume. The data confirmed that patients receiving 50 Gy (predominantly BCS cases) had a significantly larger mean PTV_Breast_ volume than those receiving 50.4 Gy (687.9 cc vs. 493.1 cc). Given that PTV_Breast_ volume was a highly significant predictor in the univariable analysis (*p* = 0.002), this suggests that the apparent effect of prescribed dose was likely confounded by PTV_Breast_ volume, a more direct anatomical risk factor. For similar reasons of collinearity, other factors such as contralateral breast volume and *D*_mean_ were also excluded from the initial multivariate model. Our multivariate analysis revealed that the risk of early-onset radiodermatitis was significantly associated with left-sided BC, low heart *V*_5_, IMRT, and large PTV_Breast_ volume.

**Fig. 2:**
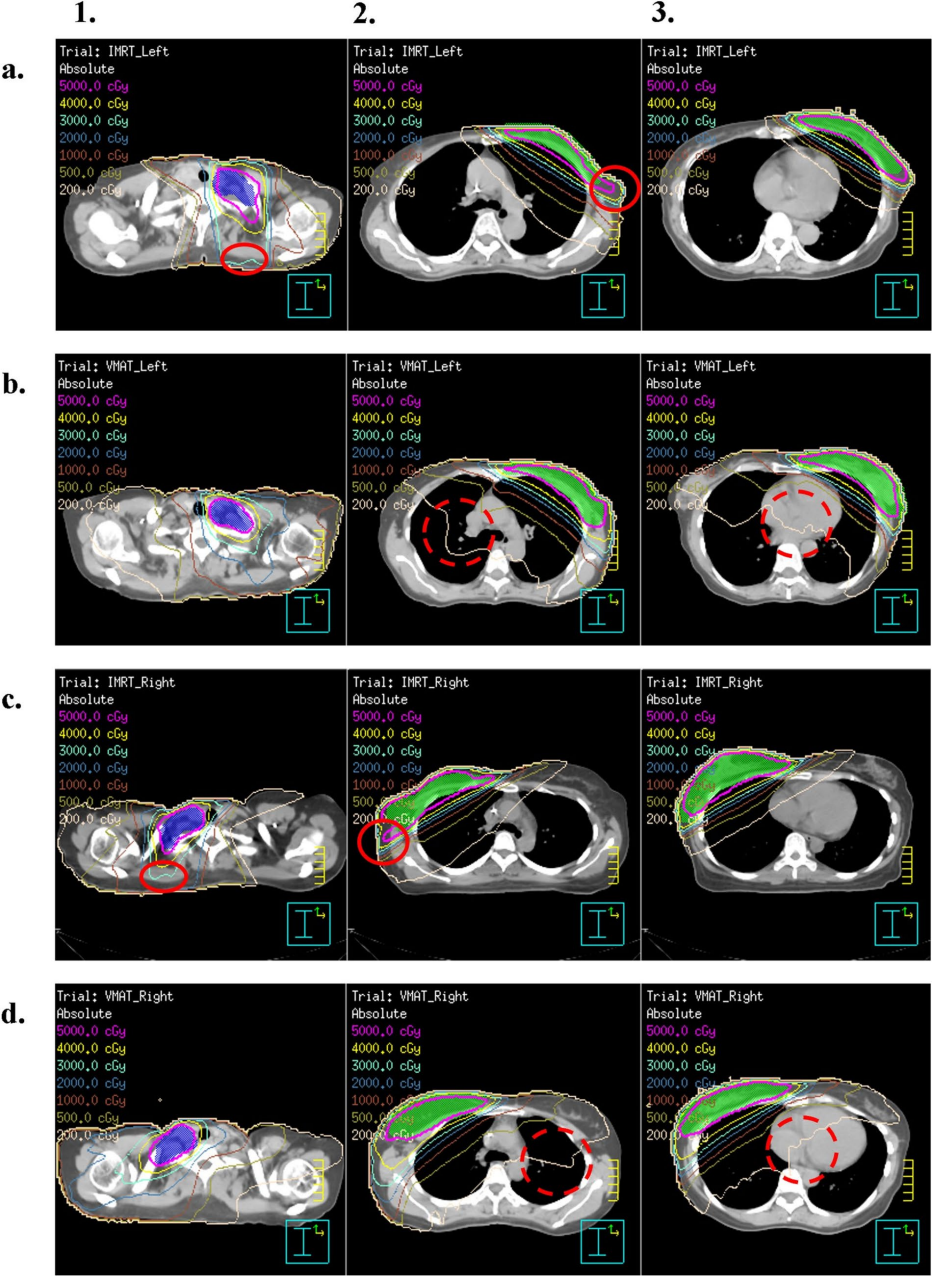
Distribution of axial doses. Axial dose distributions for a patient with left-sided breast cancer (**a** and **b**) and a patient with right-sided breast cancer (**c** and **d**) receiving IMRT (**a** and **c**) and VMAT (**b** and **d**), respectively. Columns 1–3 indicate axial slices at different levels: (1) planning target volume for the supraclavicular fossa, (2) upper breast, and (3) heart. Red solid-line circles highlight areas of dose accumulation along specific beam pathways in IMRT (**a** and **c**), whereas red dashed-line circles indicate broad low-dose distribution (200 cGy isodose curve) in VMAT (**b** and **d**). *IMRT* intensity-modulated radiotherapy, *VMAT* volumetric modulated arc therapy

The strong association between left-sided BC and an increased risk of early-onset radiodermatitis was a key finding. It is noteworthy that this appears inconsistent with our dosimetric findings for right-sided cases, where no significant differences in HI_PTVBreast_ or CI_PTVBreast_ were observed between IMRT and VMAT. This comparison of right-sided plans serves as a valuable baseline, suggesting that both techniques can achieve comparable plan quality in an anatomically unconstrained scenario. Therefore, we hypothesize that the 'left-sided' variable in our multivariable model acts as a surrogate for the entire set of planning complexities introduced by the clinical imperative to spare the heart. To minimize cardiac exposure, radiotherapy planning for left-sided BC often requires specific strategies, such as more restricted beam angulations. These strategies may lead to sharper dose gradients and inadvertently increase the radiation dose to the skin along certain pathways, creating localized high-dose regions that are not fully captured by global metrics like CI and HI. This interpretation suggests that the process of constraining a plan for cardiac sparing inherently creates a high-risk environment for the skin. The observed protective effect of high heart *V*_5_ may reflect trade-offs in dose distribution, where cardiac sparing increases skin dose along certain beam paths. This hypothesis lacks direct literature support and warrants further investigation with detailed skin dose mapping. Similarly, the IMRT-associated increase in the risk of radiodermatitis may be attributable to variations in dose distribution patterns (red solid-line circles in Fig. 2). VMAT’s advanced dose distribution capabilities across multiple angles may effectively spare the skin.

A large PTV_Breast_ volume was significantly associated with an increased risk of early-onset radiodermatitis, likely because of the higher cumulative radiation dose required to cover larger target areas. Additionally, the self-bolusing effect of the inframammary fold in large breasts may further increase this risk, leading to frequent occurrences of radiodermatitis [24]; however, the OR for early-onset radiodermatitis (1.002) suggests a minimal clinical effect per unit increase in volume.

Knowledge regarding primary risk factors for early-onset radiodermatitis may help with the implementation of targeted preventive measures, such as specialized nursing care, refined radiation planning, and early pharmacological interventions. For patients with left-sided BC receiving IMRT, clinicians should consider enhanced skin monitoring and prophylactic topical treatments starting from the first week of radiotherapy to mitigate the risk of early-onset radiodermatitis. The current strategies for managing radiodermatitis involve applying topical agents such as water-based moisturizers and creams as well as topical corticosteroids [25, 26]. Early diagnosis and proactive management are crucial for preventing severe skin toxicity and improving patient quality of life. Thus, personalized treatment plans and comprehensive supportive care are essential.

Our dosimetric analysis revealed that VMAT provided superior target coverage, as measured by V_95%_, for both PTV_Breast_ and PTV_SCF_ regardless of laterality; these findings align with those of Huang [27]. However, the superiority of VMAT in terms of conformity and homogeneity was not consistently observed across all target volumes, particularly for the PTV_Breast_ in right-sided cases. Furthermore, in patients with left-sided BC, VMAT reduced exposure of the heart and LV to high-dose radiation. Conversely, IMRT offered superior cardiac sparing in patients with right-sided BC. Therefore, an appropriate radiotherapy technique should be selected on the basis of tumor laterality to minimize the risk of radiation-induced cardiac toxicity. IMRT also exhibited superior sparing of the contralateral breast and lung, particularly in terms of low-dose exposure (red dashed-line circles in Fig. 2), reducing the risk of secondary malignancies, as reported by Karpf [28]. For the ipsilateral lung, a distinct trade-off emerged in which VMAT lowered the high-dose burden (*V*_40_) while incurring a modest yet significant increase in low-dose exposure (*V*_5_).

The differences between IMRT and VMAT likely stemmed from the difference in beam arrangements: IMRT’s tangential static beams confine the radiation path, whereas VMAT’s two ipsilateral arcs result in a relatively diffused low-dose distribution. The broad low-dose distribution in VMAT underscores the need for personalized treatment planning that balances target coverage and normal tissue sparing. Although VMAT’s superior target coverage and cardiac sparing are beneficial in patients with left-sided BC, its potential for increased low-dose spread necessitates optimization. Techniques such as tangential partial arcs [29] and knowledge-based planning [30] may improve dose distribution in VMAT. However, such modifications may inadvertently increase skin dose because of focused beam angles, similar to the angles in IMRT. Therefore, when designing personalized treatment plans, clinicians should carefully weigh each patient’s risk factors for radiodermatitis against the dosimetric advantages of each technique.

This study has several limitations that warrant consideration. First, a key limitation is its retrospective nature, wherein the choice between IMRT and VMAT was not randomized but was dependent on the treatment era. Although our multivariable analysis adjusted for known factors, we cannot exclude the possibility that other unmeasured temporal changes in patient management influenced the outcomes. Second, a major limitation is the confounding effect of DIBH. In our cohort, DIBH was used exclusively in the left-sided VMAT group, which is evidenced by the significantly larger mean ipsilateral lung volume in this group compared to the IMRT group (1330.45 cc vs. 1048.27 cc, *p* = 0.010). Consequently, the superior cardiopulmonary sparing observed, specifically the significant reductions in various heart and LV dose metrics (e.g., *D*_max_, *V*_40_, *V*_25_, *V*_10_) and in the high-dose volume to the ipsilateral lung (*V*_40_), cannot be attributed to the VMAT technique alone but to the combined effect of VMAT and DIBH.

Further limitations include the relatively small sample size, which might not have been sufficient for detecting cancer side-specific differences in risk factors, and the low incidence of Grade ≥ 2 radiodermatitis, which precluded an analysis of severe reactions. Finally, the lack of detailed spatial data on the location of skin reactions hindered investigation of its correlation with dose distribution. Future large-scale studies involving comprehensive skin reaction mapping and standardized application of techniques like DIBH should be conducted to address these limitations.

## Conclusion

We identified left-sided BC, large PTV_Breast_ volume, and IMRT as key risk factors for early-onset radiodermatitis in patients with locally advanced unilateral BC, while high heart *V*_5_, indicating greater exposure of the heart to low-dose radiation, was associated with reduced risk. Furthermore, our dosimetric analysis revealed distinct advantages and disadvantages of VMAT and IMRT. VMAT exhibited superior tumor coverage and cardiac sparing, particularly in patients with left-sided BC. In contrast, IMRT minimized low-dose exposure to the contralateral breast and lung. Therefore, while designing personalized treatment plans, clinicians should carefully consider individual patient characteristics and dosimetric parameters to optimize tumor control and minimize the risk of radiation-induced toxicity. Future studies should explore spatial skin dose distribution and conduct multi-center trials to validate these findings and refine personalized radiotherapy strategies.

## Data Availability

The datasets generated and/or analysed during the current study are not publicly available due to patient privacy and ethical restrictions but are available from the corresponding author on reasonable request.

## Declarations

### Trial registration

This study was approved by the Joint Institutional Review Board of Taipei Medical University, Taiwan (Permit number: N201912025).
